# Deletion of 18q is a strong and independent prognostic feature in prostate cancer

**DOI:** 10.18632/oncotarget.13404

**Published:** 2016-11-16

**Authors:** Martina Kluth, Maximilian Graunke, Christina Möller-Koop, Claudia Hube-Magg, Sarah Minner, Uwe Michl, Markus Graefen, Hartwig Huland, Raisa Pompe, Frank Jacobsen, Andrea Hinsch, Corinna Wittmer, Patrick Lebok, Stefan Steurer, Franziska Büscheck, Till Clauditz, Waldemar Wilczak, Guido Sauter, Thorsten Schlomm, Ronald Simon

**Affiliations:** ^1^ Institute of Pathology, Prostate Cancer Center at University Medical Center Hamburg-Eppendorf, Germany; ^2^ Martini-Clinic, Prostate Cancer Center at University Medical Center Hamburg-Eppendorf, Germany; ^3^ Department of Urology, Section for prostate cancer research, University Medical Center Hamburg-Eppendorf, Germany

**Keywords:** 18q deletion, prostate cancer, prognosis, tissue microarray

## Abstract

Deletion of 18q recurrently occurs in prostate cancer. To evaluate its clinical relevance, dual labeling fluorescence *in-situ* hybridization (FISH) using probes for 18q21 and centromere 18 was performed on a prostate cancer tissue microarray (TMA). An 18q deletion was found in 517 of 6,881 successfully analyzed cancers (7.5%). 18q deletion was linked to unfavorable tumor phenotype. An 18q deletion was seen in 6.4% of 4,360 pT2, 8.0% of 1,559 pT3a and 11.8% of 930 pT3b-pT4 cancers (*P* < 0.0001). Deletions of 18q were detected in 6.9% of 1,636 Gleason ≤ 3 + 3, 6.8% of 3,804 Gleason 3 + 4, 10.1% of 1,058 Gleason 4+3, and 9.9% of 344 Gleason ≥ 4 + 4 tumors (*P* = 0.0013). Deletions of 18q were slightly more frequent in ERG-fusion negative (8.2%) than in ERG-fusion positive cancers (6.4%, *P* = 0.0063). 18q deletions were also linked to biochemical recurrence (BCR, *P* < 0.0001). This was independent from established pre- and postoperative prognostic factors (*P* ≤ 0.0004). In summary, the results of our study identify 18q deletion as an independent prognostic parameter in prostate cancer. As it is easy to measure, 18q deletion may be a suitable component for multiparametric molecular prostate cancer prognosis tests.

## INTRODUCTION

Prostate cancer is one of the most frequent cancer types in males worldwide. About one million patients are diagnosed with this disease every year, and almost 140,000 eventually die from their cancer in Western societies [1]. Autopsy studies suggest that up to 70% of males will develop prostate cancer during their lifetime, but clinical experience shows that only a minority of patients will develop life-threatening disease that requires radical treatment [2, 3]. As screening strategies identify prostate cancers already at early stages of the disease, it becomes increasingly important to avoid overtreatment of patients with less aggressive disease. Accordingly, it will be important to establish molecular markers enabling an early distinction between the indolent and aggressive forms of the disease.

Chromosomal deletions are a hallmark of prostate cancer [4–7]. These deletions occur of variable size at multiple chromosomal loci, including for example 3p13, 17p13, 5q21, 6q15 and the PTEN locus at 10q23, all of which have been associated with adverse histological features and poor clinical outcome [5–12]. Little is known about the clinical impact of other slightly less frequent deletions in prostate cancer, including deletions involving large parts of chromosome 18q. Published data from gene copy number screening studies employing classical or array-based comparative genomic hybridization [5, 7, 13–16] suggest that this alteration may occur in 3% and 37% of prostate cancers. Earlier studies analyzing loss of heterozygosity (LOH) in small cohorts of 23–46 prostate cancers suggested that 18q loss might be linked to advanced stage [17, 18] or high Gleason grade [18]. Also considering that 18q harbors multiple genes with key roles in several cancers types, including for example the deleted in colon cancer (DCC, 18q21.2) tumor suppressor [19], or the B-cell lymphoma (BCL2, 18q21.33) apoptosis regulator [20], 18q deletion is of potential biological relevance in prostate cancers.

In order to clarify the prevalence and clinical significance of 18q deletions, we performed fluorescence in-situ hybridization (FISH) analysis with an 18q21-specific FISH probe in more than 12,000 prostate cancers in a tissue microarray (TMA) format.

## RESULTS

### Technical aspects

18q FISH analysis was successful in 6,881 of 12,247 (56.2%) arrayed cancers. Analysis was not informative in the remaining 5,366 tumors because of lack of tumor cells in the tissue spots, faint or absent FISH signals, or missing tissue spots on the TMA section.

### 18q deletions and prostate cancer phenotype

Heterozygous 18q deletions were found in 7.5% (517/6,881) of all prostate cancers. Homozygous 18q deletions were not observed. 18q deletions were significantly linked to advanced tumor stage (*P* < 0.0001), high Gleason grade (*P* = 0.0013) and presence of tumor in the surgical margin (*P* = 0.0416). All results on 18q deletions and prostate cancer phenotype are summarized in Table 1.

**Table 1:** Clinico-pathological association of 18q deletion in all cancers, ERG-negative cancers and ERG-positive cancers

	All cancers			ERG-negative cancers			ERG-positive cancers		
	*n*	18q deletion (%)	*p* value	*n*	18q deletion (%)	*p* value	*n*	18q deletion (%)	*p* value
**All cancers**	6881	7.5		3419	8.2		2977	6.4	
**Tumor stage**									
pT2	4360	6.4	< 0.0001	2260	6.9	0.0004	1751	5.1	< 0.0001
pT3a	1559	8.0		687	9.8		790	6.5	
≥ pT3b	930	11.8		459	12.0		418	11.2	
**Gleason grade**									
≤ 3+3	1636	6.9	0.0013	799	7.9	0.1842	667	5.0	0.0011
3+4	3804	6.8		1826	7.5		1745	5.6	
4+3	1058	10.1		569	10.2		428	10.3	
≥ 4+4	344	9.9		208	9.6		116	10.3	
**PSA Level (ng/μl)**									
< 4	863	8.0	0.0630	377	7.4	0.3525	413	8.2	0.0698
4–10	4035	6.7		1961	7.7		1784	5.5	
10–20	1383	8.8		756	9.7		539	6.7	
> 20	510	8.4		289	7.3		195	9.2	
**Lymph node metastasis**									
N0	3905	7.4	0.2688	1973	7.7	0.1854	1690	6.9	0.8311
N+	400	9.0		191	10.5		186	6.5	
**Surgical margin**									
negative	5390	7.2	0.0416	2694	8.0	0.3797	2300	5.9	0.0581
positive	1366	8.9		664	9.0		620	8.1	

### 18q deletion and ERG fusion status

**Figure 1:**
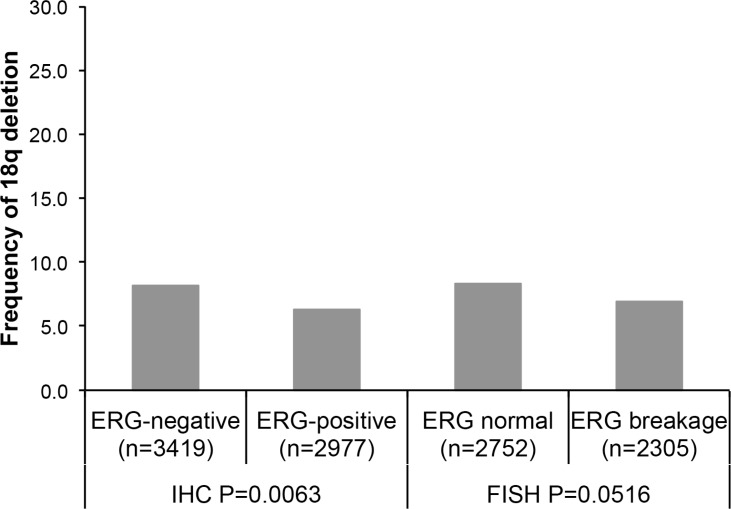
Associations between 18q deletion and ERG-fusion by IHC and FISH analysis

18q deletions were marginally more frequent in ERG-negative cancers irrespective of the method of ERG analysis (*P* = 0.0063 for ERG-IHC and *P* = 0.0516 for ERG-FISH analysis). Deletions of 18q were found in 8.2% and 8.4% ERG-negative cancers (according to ERG IHC and FISH analysis), and in 6.4% (IHC) and 6.9% (FISH) ERG-positive cancers (Figure 1). Statistical associations of 18q deletions and tumor phenotype were weaker in the subsets of ERG-positive and ERG-negative cancers, or vanished completely (Table 1). This phenomenon may be due to smaller numbers in the analysis of a relatively rare event (< 10%).

### 18q deletions and clinical outcome

**Figure 2:**
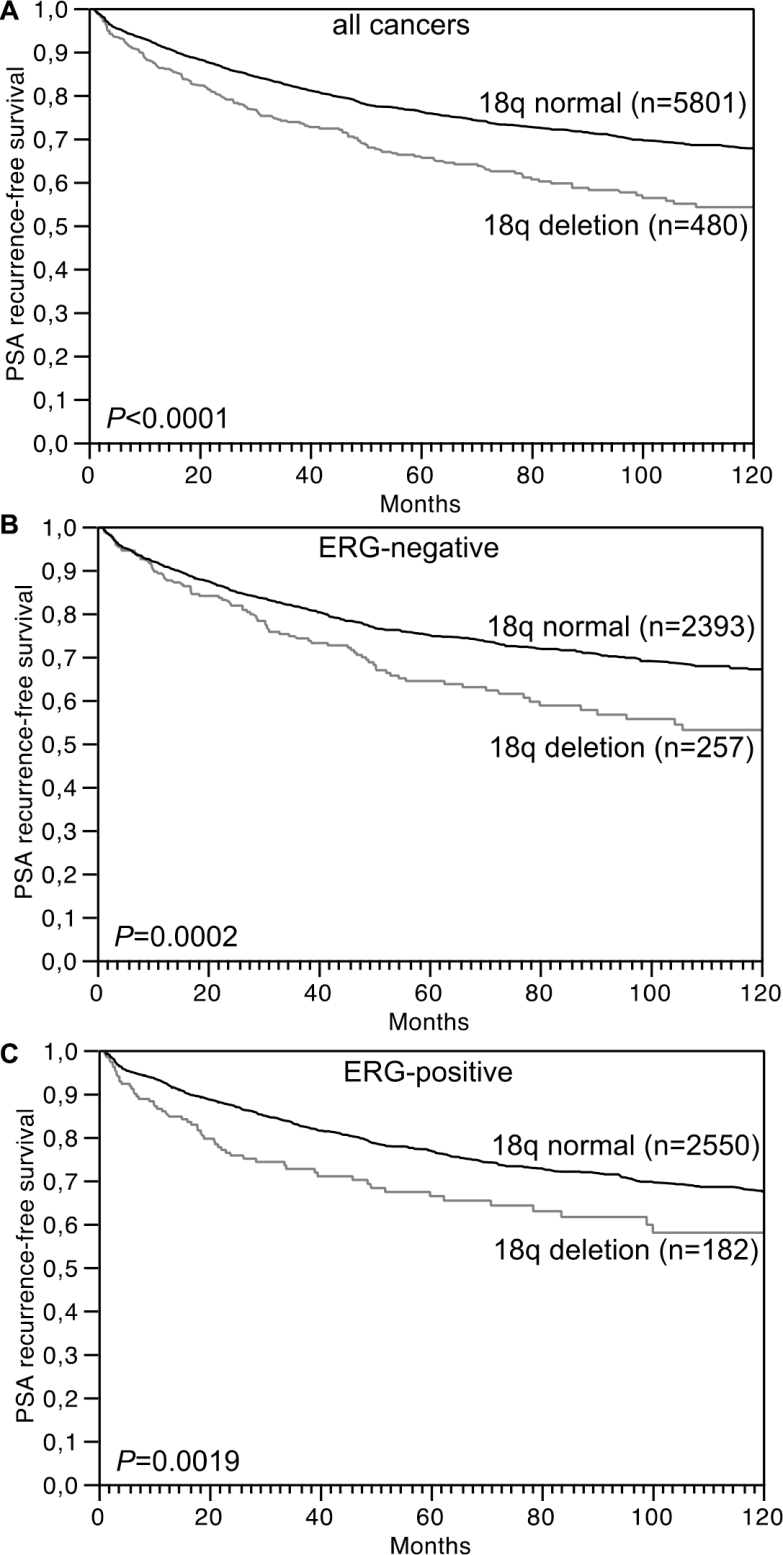
Association between 18q deletion and biochemical (PSA) recurrence in (A) all cancers (*n* = 6,281), (B) ERG-fusion negative cancers (*n* = 2,650) and (C) ERG-fusion positive cancers (*n* = 2,732)

**Figure 3:**
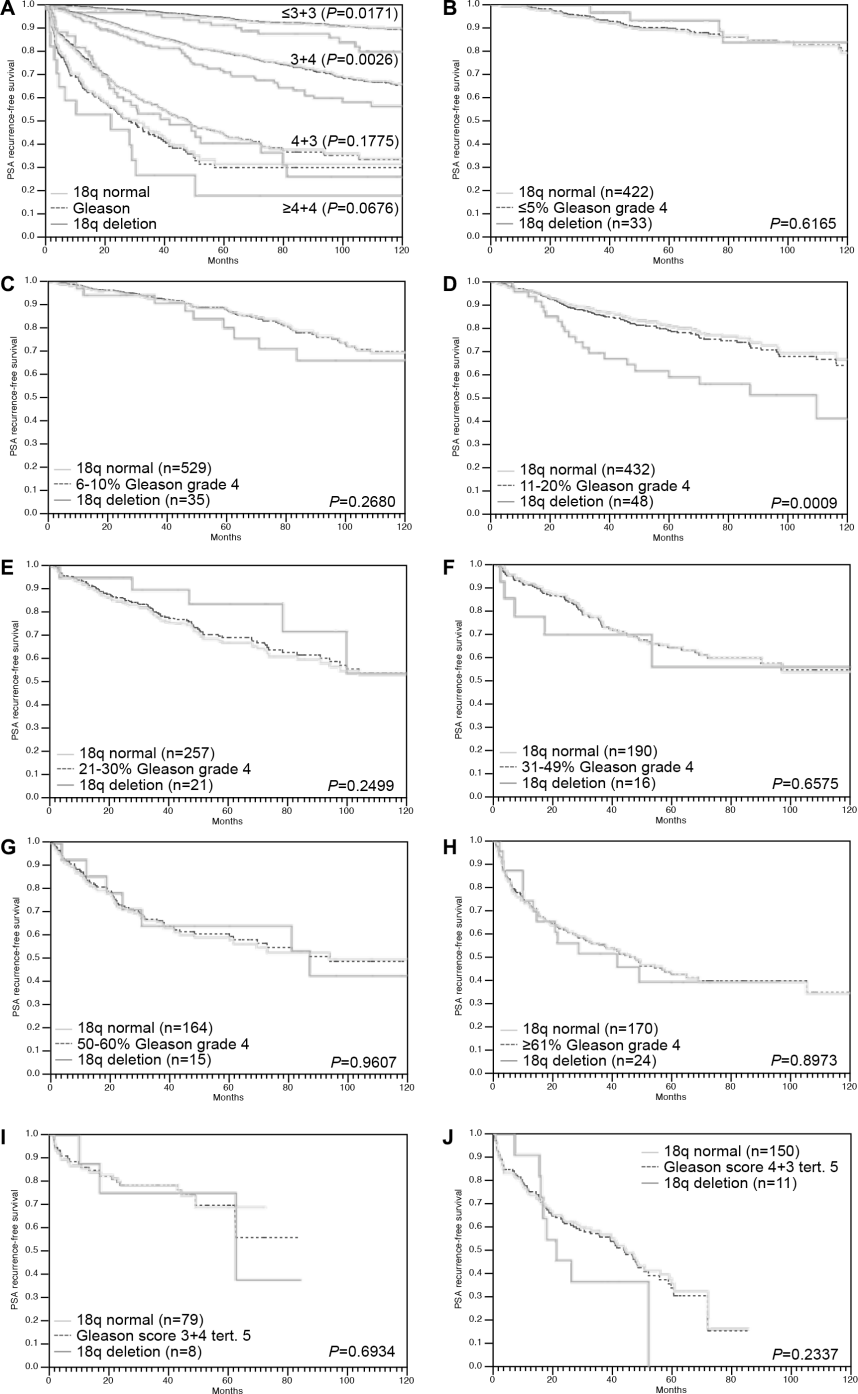
Association between 18q deletion and biochemical recurrence in dependence on (A) Gleason Grade (*n* = 1,535 for ≤ 3 + 3, *n* = 3,430 for 3 + 4, *n* = 984 for 4 + 3 and *n* = 323 for ≥ 4 + 4) and (B–J) quantitative Gleason grading subgroups

Follow-up data were available from 6,281 tumors that were successfully analyzed for 18q deletion. In univariate analysis, 18q deletions were strongly linked to biochemical (PSA) recurrence in all cancers (*P* < 0.0001, Figure 2A) as well as in the subsets of 2,650 ERG-negative (*P* = 0.0002, Figure 2B) and 2,732 ERG-positive cancers (*P* = 0.0019, Figure 2C). Moreover, 18q deletion provided additional prognostic impact in low and intermediate risk groups including Gleason 3 + 3 = 6 (*P* = 0.0171), and Gleason 3 + 4 = 7 (*P* = 0.0026). 18q deletion did not add further prognostic information in high grade cancers (≥ 4 + 3; Figure 3A). Furthermore, 18q deletions were not prognostically relevant in subgroups of tumors with comparable quantitative Gleason score, with the only exception of cancers with 11–20% Gleason 4 (Figure 3B–3H).

### Multivariate analyses

The prognostic relevance of 18q deletion was further assessed in four different multivariate analyses, including established pre- and postoperative prognostic parameters. Scenario 1 included preoperative PSA value, the postoperative parameters pathological tumor stage (pT), pathological lymph node status (pN), surgical margin status (R) and pathological Gleason grade obtained on the entire resected prostate as well as 18q deletion status. Scenario 2 evaluated 18q deletion status, preoperative PSA value and all postoperative parameters with exception of nodal status. The rational for this approach was that indication and extent of lymph node dissection is not standardized in the surgical therapy of prostate cancer and that excluding pN in multivariate analysis can markedly increase case numbers. Scenario 3 included 18q deletion status, preoperative PSA value, clinical tumor stage (cT) and Gleason grade obtained on the prostatectomy specimen. It is of note, that postoperative determination of a tumors Gleason grade is usually “better” than the preoperatively determined Gleason grade (subjected to sampling errors and consequently under-grading in more than one third of cases [21]). Therefore, in scenario 4, the preoperative Gleason grade obtained on the original biopsy was combined with preoperative PSA value, cT and 18q deletion status. All multivariate analyses revealed that 18q deletion predicted PSA recurrence independently in all scenarios (*P* ≤ 0.0004) and that the predictive value of 18q deletion was largely independent of the ERG status (*P* ≤ 0.02 in ERG-negative and *P* ≤ 0.065 in ERG-positive cancers, Table 2).

**Table 2:** Multivariate analysis (Cox regression) including clinical and pathological parameters in addition to 18q deletion in all cancers, ERG negative cancers and ERG positive cancers

Tumor subset	Scenario	n analyzable	P value							
			preop. PSA-Level	pT Stage	cT Stage	Gleason grade prostatectomy	Gleason grade biopsy	pN Stage	R-Status	18q Deletion
all cancers	1	3819	< 0.0001	< 0.0001		< 0.0001		< 0.0001	0.0097	0.0004
	2	6126	< 0.0001	< 0.0001		< 0.0001			< 0.0001	0.0004
	3	6008	< 0.0001		< 0.0001	< 0.0001				0.0001
	4	5925	< 0.0001		< 0.0001		< 0.0001			< 0.0001
ERG-negative cancers	1	1916	< 0.0001	< 0.0001		< 0.0001		0.0001	0.0749	0.0148
	2	3030	< 0.0001	< 0.0001		< 0.0001			0.0024	0.0157
	3	2988	< 0.0001		< 0.0001	< 0.0001				0.0067
	4	2949	< 0.0001		< 0.0001		< 0.0001			0.0024
ERG-positive cancers	1	1674	0.0282	< 0.0001		< 0.0001		0.0355	0.1069	0.0264
	2	2666	0.0009	< 0.0001		< 0.0001			0.0015	0.0634
	3	2598	< 0.0001		< 0.0001	< 0.0001				0.0335
	4	2560	< 0.0001		< 0.0001		< 0.0001			0.0593

## DISCUSSION

The results of our study identify 18q deletion as a strong predictor of poor prognosis in prostate cancer.

About 7% of the 6,881 prostate cancers analyzed in our study showed heterozygous deletions of 18q. This is in the lower range of previous high-resolution array-based comparative genomic hybridization (aCGH) studies reporting 18q deletions in 3–32% of tumors [5, 7, 22–24]. Higher deletion frequencies were reported from studies employing less quantitative methods such as conventional comparative genomic hybridization (CGH; 21–37%) [13–16] or loss of heterozygosity (LOH) analysis (10–45%) [17, 18, 25–31]. FISH is regarded as the most precise method for gene copy number determination as it allows for analysis on the single cell level and is not disturbed by contaminating non-neoplastic cell that are inevitably present in cancer tissues. Scoring criteria for our deletion analysis in tissue sections had earlier been validated by comparison of FISH and array CGH data [10] and had been successfully used to analyzed other deletions in our prognosis TMA [8–12]. A FISH probe targeting the NEDD4L (neuronal precursor cell expressed, developmentally down-regulated 4-like, 18q21) gene had been selected because it maps to the center of the often large 18q deletions in prostate cancer [7] and it is one of the candidate tumor suppressor genes located on 18q. Other examples of established or suggested 18q tumor suppressor genes include deleted in colon cancer (DCC) gene (18q21.2) [19], PH domain and leucine rich repeat protein phosphatase 1 (PHLPP1) gene (18q21.33) [32], serpin peptidase inhibitor (SERPINB5) gene (18q21.33) [33], Desmoglin 2 (DSG2) gene (18q12.1) [34], retinoblastoma binding protein (RBBP8) gene (18q11.2) [35], suppressor of cytokine signaling 6 (SOCS6) gene (18q22) [36], and a cluster of important genes involved in the regulation of canonical TGFß-signaling, including the SMAD family members 2 and 4 (SMAD2, 18q21.1 and SMAD4, 18q21.2) [37].

Deletions of 18q were significantly associated with advanced tumor stage, high Gleason grade and increased risk of biochemical recurrence. These findings are in line with earlier studies on 23–46 prostate cancers reporting higher frequencies of LOH at 18q21 in tumors with advanced stage [17, 18], high Gleason grade and metastatic growth [26]. That the prognostic impact of 18q deletions was independent of established prognostic features, both in preoperative and in postoperative scenarios, highlights the potential clinical applicability of 18q deletion measurement. The Gleason grade is the strongest known prognostic parameter in prostate cancer. A comparison with established prognostic Gleason grade groups revealed, that 18q deletion was even able to distinguish prognostic subgroups within Gleason 3 + 3 and 3 + 4 cancers. This is of particular interest because these are the low and intermediate risk tumors where the clinical decison making is most difficult and where the therapeutic options often range from active surveillance to radical prostatectomy.

Based on the morphologic analysis of more than 10,000 prostate cancers, we had recently shown, that prognostic Gleason Grade information can be expanded by using the percentage of unfavorable Gleason patterns as a continuous variable. Both in biopsies and in prostatectomy samples, prostate cancer prognosis continuously deteriorates with increasing percentage of unfavorable Gleason pattern found in a cancer (quantitative Gleason Grade) [38]. The lack of an unequivocal prognostic impact of 18q deletion in most subgroups defined by a comparable quantitative Gleason grade illustrates how difficult it is for a molecular parameter to outperform established morphological parameters of malignancy. However, a variety of molecular features have recently been identified [4, 8–12, 39, 40]. We thus anticipate, that a clinically useful molecular prostate cancer prognosis test combining a number of strong prognostic biomarkers will become feasible. Because of the simple and reproducible analysis of 18q deletions always resulting in a yes/no answer, we believe that 18q deletions could be a suitable element for such a test.

Almost all chromosomal deletions occurring at relevant frequency are strongly linked to either ERG-positive or ERG-negative prostate cancers. For example, deletions of 6q15 and 5q21 are frequent in ERG fusion negative cancers [7, 9, 12], whereas deletions of 3p13, 16q23, TP53 and PTEN are common in ERG fusion positive cancers [7, 8, 10, 11, 39, 41, 42]. Remarkably, all strongly ERG-associated chromosomal deletions are prognostically relevant [8–12, 39], while the ERG status by itself is completely unrelated to clinical outcome [43]. ERG is a transcription factor, which becomes expressed as a result of a gene fusion involving the androgen-regulated gene *TMPRSS2* and the *ERG* locus in about 50% of prostate cancers [7, 43, 44]. ERG fusion is an early event in prostate cancer that triggers paramount changes of the cancer cell micro-environment, some of which may impact the likelihood for development of certain deletions. It is of note, that our data do not show substantial differences in the 18q deletion rate between ERG-negative and ERG-positive cancers. It is thus likely, that none of the genes affected by common 18q deletions is influencing possible mechanisms needed for the development of *TMPRSS2*:*ERG* fusion.

The lack of homozygous 18q deletions in our study and the absence of recurrent mutations of these genes in deep sequencing studies involving almost 500 prostate cancers (http://www.cbioportal.org [45, 46]) argue against a biallelic inactivation of 18q genes by either homozygous deletions or deletion of one allele and mutation of the other allele. These findings challenge the classical recessive model of biallelic tumor suppressor gene inactivation but may also offer novel therapeutic options. Heterozygous deletion of essential genes has been postulated to render cancer cells vulnerable to further inhibition of these genes, and 56 genes have been identified until now suppression of which specifically inhibited the proliferation of cells harboring partial copy number loss of these genes [47]. Such essential genes had been suggested as promising targets for anti-cancer therapies, and were thus termed CYCLOPS (copy number alterations yielding cancer liabilities owing to partial loss) genes [47]. Given the large size of 18q deletions involving more than 200 genes it seems likely that also 18q harbors essential genes, some of which might represent promising candidates for potential targeted therapies.

In summary, the results of our study demonstrate a strong and independent prognostic impact of 18q deletions in prostate cancer. As 18q deletions are easy to analyze and the analyses provide a simple and highly reproducible yes/no answer, this parameter appears to highly suitable as an element for a multiparametric clinically applicable prostate cancer prognosis test.

## MATERIALS AND METHODS

### Patients

Radical prostatectomy specimens were available from 12,427 patients, undergoing surgery between 1992 and 2012 at the Department of Urology and the Martini Clinic at the University Medical Center Hamburg-Eppendorf. Histo-pathological data was retrieved from the patient files, including tumor stage, Gleason grade, nodal stage and stage of the resection margin. In addition to the classical Gleason categories, “quantitative” Gleason grading was performed as described before [38]. In brief, for every prostatectomy specimen, the percentages of Gleason 4 patterns in cancerous tissues were estimated during the regular process of pathologic interpretation. Gleason 3+4 and 4+3 cancers were subdivided according to their percentage of Gleason 4. For practical use, we subdivided the 3+4 and 4+3 cancers in 8 subgroups: 3+4 ≤ 5% Gleason 4, 3+4 6–10%, 3+4 11–20%, 3+4 21–30%, 3+4 31–49%, 4+3 50–60%, 4+3 61–80% and 4+3 > 80% Gleason 4. In addition, separate groups were defined by the presence of a tertiary Gleason 5 pattern, including 3+4 Tert. 5 and 4+3 Tert. 5. Follow-up data were available for a total of 11,665 patients with a median follow-up of 36 months (range: 1 to 241 months; Table 3). Prostate specific antigen (PSA) values were measured following surgery and PSA recurrence was defined as the time point when postoperative PSA was at least 0.2 ng/ml and increasing at subsequent measurements. All prostate specimens were diagnosed according to a standard procedure, including complete embedding of the entire prostate for histological analysis [48]. The TMA manufacturing process was described earlier in detail [49]. In short, one 0.6 mm core was taken from a representative tissue block from each patient. The tissues were distributed among 10 TMA blocks, each containing 144 to 522 tumor samples. Each TMA block also contained various control tissues, including normal prostate tissue. The molecular database attached to this TMA includes data on ERG expression in 6,396 and on *ERG* rearrangement by FISH analysis in 5,057 (extended from [4, 43]) cancers. Analysis of patient and corresponding histopathological data for research purposes, as well as construction of tissue microarrays from archived diagnostic left-over tissues, was approved by local laws (HmbKHG, §12,1) and by the local ethics committee (Ethics commission Hamburg, WF-049/09 and PV3652). All work was carried out in compliance with the Helsinki Declaration.

**Table 3:** Composition of the prognosis tissue microarray containing 12,427 prostate cancer specimens

	No. of patients (%)	
	Study cohort on TMA	Biochemical relapse among categories
	(*n* = 12427)	
**Follow-up (mo)**		
n	11665 (93.9%)	2769 (23.7%)
Mean	48.9	−
Median	36.4	−
**Age (y)**		
≤ 50	334 (2.7%)	81 (24.3%)
51–59	3061 (24.8%)	705 (23.0%)
60–69	7188 (58.2%)	1610 (22.4%)
≥ 70	1761 (14.3%)	370 (21.0%)
**Pretreatment PSA (ng/ml)**		
< 4	1585 (12.9%)	242 (15.3%)
4–10	7480 (60.9%)	1355 (18.1%)
10–20	2412 (19.6%)	737 (30.6%)
> 20	812 (6.6%)	397 (48.9%)
**pT category (AJCC 2002)**		
pT2	8187 (66.2%)	1095 (13.4%)
pT3a	2660 (21.5%)	817 (30.7%)
pT3b	1465 (11.8%)	796 (54.3%)
pT4	63 (0.5%)	51 (81.0%)
**Gleason grade**		
≤ 3+3	2983 (24.1%)	368 (12.3%)
3+4	6945 (56.2%)	1289 (18.6%)
4+3	1848 (15.0%)	788 (42.6%)
≥ 4+4	584 (4.7%)	311 (53.3%)
**pN category**		
pN0	6970 (91.0%)	1636 (23.5%)
pN+	693 (9.0%)	393 (56.7%)
**Surgical margin**		
Negative	9990 (81.9%)	1848 (18.5%)
Positive	2211 (18.1%)	853 (38.6%)

NOTE: Numbers do not always add up to 12,427 in the different categories because of cases with missing data. Abbreviation: AJCC, American Joint Committee on Cancer.

### Fluorescence in-situ hybridization

**Figure 4:**
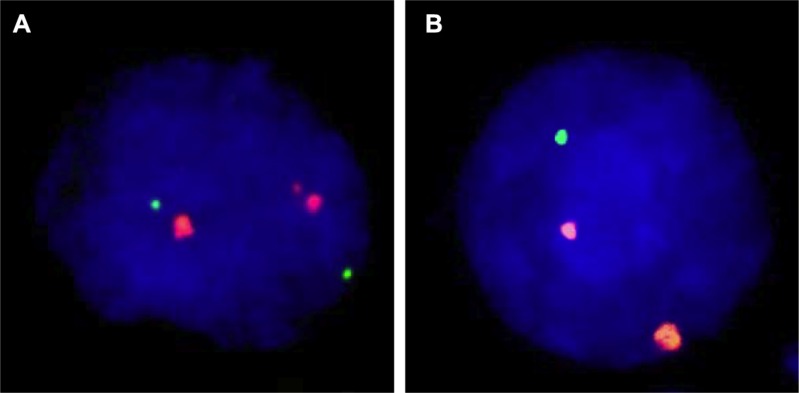
Examples of FISH findings using the 18q deletion probe (**A**) Normal 18q copy numbers as indicated by two green 18q signals and two orange centromere 18 signals. (**B**) Heterozygous deletion as indicated by the lack of one green 18q signal.

Four micrometer TMA sections were used for fluorescence in-situ hybridization (FISH). TMA sections were de-waxed, air-dried, and dehydrated in 70%, 85%, and 100% ethanol. Slides were pretreated in VP 2000 Pretreatment Reagent (Abbott, Des Plaines, USA) for 15 min at 80°C, followed by 150 min incubation at 37°C in 0.5% protease 1 solution (Abbott, Des Plaines, USA). 4 μl of FISH probe mix in 70% formamide 2x SSC solution was applied to the slides and co-denatured with the cellular DNA in a Hybrite hybridization oven for 10 min at 72°C prior to overnight-hybridization at 37°C in a humidified chamber. The FISH probe mix consisted of a spectrum-green labeled 18q (NEDD4L locus, 18q21.31) probe (made from BACs RP11-167O10 and BAC RP11-718I15), and a spectrum-orange labeled, commercial centromere 18 probe (#5J08-18; Abbott, Wiesbaden, Germany) as a reference. After hybridization, slides were subjected to serial stringent washings (2× SSC solution with 0.3% NP40 at 72°C for 2 minutes) and counterstained with 0.2 μmol/L 4’-6-diamidino-2-phenylindole (DAPI) in antifade solution. Stained slides were manually interpreted under an epifluorescence microscope, and the predominant green and orange FISH signal numbers were recorded in each tissue spot. Homozygous deletion of 18q was defined as complete lack of 18q FISH signals in the tumor nuclei, but presence of 18q FISH signals in adjacent normal cells. Tissue spots lacking 18q signals in all (tumor and normal cells), or lacking of any normal cells as an internal control for successful hybridization of the 18q probe, were excluded from analysis. Heterozygous deletion of 18q was defined as the presence of fewer 18q signals than centromere 18 probe signals of ≥ 60% tumor nuclei. These thresholds were based on a previous study analyzing PTEN deletions in a subset of slides of the TMA set [50]. Representative FISH images are shown in Figure 4.

### Statistics

For statistical analysis, the JMP software (SAS Institute Inc., NC, USA) was used. Contingency tables were calculated to study association between 18q deletion and clinico-pathological parameters, and the Chi-square (Likelihood) test was used to find significant relationships. Kaplan Meier plots were generated for PSA recurrence-free survival. The Log-Rank test was applied to determine the significance of differences between the survival curves. Cox proportional hazards regression analysis was performed to test the statistical independence and significance between pathological, molecular, and clinical variables.
